# Adherence to low-dose methotrexate in children with juvenile idiopathic arthritis using a sensitive methotrexate assay

**DOI:** 10.1186/s12969-024-00988-y

**Published:** 2024-05-07

**Authors:** Julia E. Möhlmann, Sytze de Roock, Annelies C. Egas, Evelien ter Weijden, Martijn J. H. Doeleman, Alwin D. R. Huitema, Matthijs van Luin, Joost F. Swart

**Affiliations:** 1 Department of Clinical Pharmacy, University Medical Centre Utrecht, Utrecht University, Utrecht, The Netherlands; 2 Department of Pediatric Rheumatology and Immunology, Wilhelmina Children’s Hospital, Utrecht University, Utrecht, The Netherlands; 3 Department of Clinical Diagnostics, University Medical Centre Utrecht, Utrecht University, Utrecht, The Netherlands; 4 Department of Pharmacy and Pharmacology, Antoni van Leeuwenhoek hospital, Netherlands Cancer Institute, Amsterdam, The Netherlands; 5 Department of Pharmacology, Princess Máxima Centre for Pediatric Oncology, Utrecht, The Netherlands

**Keywords:** Methotrexate, MTX, Juvenile idiopathic arthritis, JIA, Drug adherence, Adherence assay

## Abstract

**Background:**

Low-dose weekly methotrexate (MTX) is the mainstay of treatment in juvenile idiopathic arthritis. Unfortunately, a substantial part of patients has insufficient efficacy of MTX. A potential cause of this inadequate response is suboptimal drug adherence. The aim of this study was to assess MTX adherence in juvenile idiopathic arthritis patients by quantification of MTX concentrations in plasma. Secondly, the association between MTX concentrations and either self-reported adherence issues, or concomitant use of biologics was examined.

**Methods:**

This was a retrospective, observational study using plasma samples from juvenile idiopathic arthritis patients. An ultrasensitive liquid chromatography-tandem mass spectrometry method was developed for quantification of MTX and its metabolite 7-hydroxy-MTX in plasma. The determined MTX plasma concentrations in juvenile idiopathic arthritis patients were compared with corresponding adherence limits, categorising them as either adherent or possibly non-adherent to MTX therapy.

**Results:**

Plasma samples of 43 patients with juvenile idiopathic arthritis were analysed. Adherence to MTX in this population was 88% shortly after initiation of MTX therapy and decreased to 77% after one year of treatment. Teenagers were more at risk for non-adherence (*p* = 0.002). We could not find an association between MTX adherence with either self-reported adherence issues, nor with the use of concomitant biological treatment (*p* = 1.00 and *p* = 0.27, respectively; Fisher’s Exact).

**Conclusions:**

Quantification of MTX in plasma is a feasible and objective method to assess adherence in patients using low-dose weekly MTX. In clinical practice, the use of this method could be a helpful tool for physicians to refute or support suspicion of non-adherence to MTX therapy.

**Supplementary Information:**

The online version contains supplementary material available at 10.1186/s12969-024-00988-y.

## Background

Juvenile idiopathic arthritis (JIA) is a chronic disease characterised by persistent joint inflammation and has its onset in children younger than 16 years. The mainstay of treatment is low-dose methotrexate (MTX), a disease-modifying antirheumatic drug (DMARD), in a weekly dose of mostly 10–15 mg/m^2^ in children with JIA [1]. Unfortunately, efficacy of MTX is insufficient in 30 to 70% of JIA patients and addition of more costly biologicals is required to reach inactive disease and prevent joint damage [2]. A potential cause of inadequate response to MTX is suboptimal drug adherence [3]. 

Adherence to MTX has been studied in JIA patients using questionnaires completed by children and parents [4, 5]. Estimated adherence percentages in JIA patients were 76 to 92% and there was an inverse association between age and adherence. A currently used and validated questionnaire in JIA patients is the Juvenile Arthritis Multidimensional Assessment Report (JAMAR) [6]. The JAMAR also contains a few questions about medication intake, which may reveal adherence issues. However, self-reported assessments or diaries are subjective methods to assess adherence, possibly overestimating true adherence percentages. This is suspected since it is known that approximately 75% of JIA patients and parents experience at least one adherence barrier concerning MTX intake [7]. Concerns about side-effects, long-term toxicities and therapy-related shame were the most commonly reported adherence barriers. Moreover, intolerance to MTX is high among JIA patients [8]. The prevalence of MTX intolerance was found to be approximately 50% according to the MTX Intolerance Severity Score (MISS) questionnaire. Both adherence barriers and intolerance issues may contribute to suboptimal adherence to MTX therapy.

Other methods to assess adherence are the use of pharmacy dispensing records and the monitoring of pill bottle openings [3]. These two indirect methods may also give an overestimation of true adherence percentages. A more direct and objective method to assess adherence could be the quantification of drug concentrations in blood. This has been shown a feasible marker for adherence to hydroxychloroquine treatment in patients with systemic lupus erythematosus [9]. Moreover, openly screening of drug concentrations in blood has been associated with better treatment outcome and improvement in therapy adherence [10]. 

Bluett et al. developed a MTX adherence assay for adults with rheumatoid arthritis (RA) using liquid chromatography tandem-mass spectrometry (LC-MS/MS) [11]. With the defined adherence limits, they were able to detect adherence in > 80% of compliant adult RA patients at 7 days after MTX administration for each dose ≥ 5 mg/week.

To date, there is no such direct and objective method available for JIA patients. Monitoring of MTX concentrations in children with JIA could be a helpful tool for physicians to identify patients in which insufficient response is probably related to non-adherence. Therefore, we developed an ultrasensitive LC-MS/MS assay to quantify both MTX and its major metabolite 7-hydroxy-MTX (7-OH-MTX) in plasma. The aim of this study was to investigate MTX adherence in children with JIA using this LC-MS/MS assay.

## Methods

### Study design and setting

We conducted a retrospective observational study using plasma samples from the Pharmachild biobank, Wilhelmina Children’s Hospital Utrecht, The Netherlands. This biobank contains blood samples of outpatient clinical visits of the Utrecht Pharmachild cohort, drawn for the purpose of studying long-term safety and efficacy of drug treatment in this population. In compliance with ethical standards, patient materials were stored after written informed consent was acquired. Ethical approvals by the institutional Medical Ethics Committee of Utrecht were obtained under protocol numbers 11-499c and 14–684. Patient demographics, MTX dose and duration of therapy, use of comedication, and reported JAMAR questionnaires were extracted from the Pharmachild registry cohort and electronic health records. It was not appropriate to involve patients in the design or conduct of our research, because it concerned a retrospective biobank research.

### Study population and sample selection

The source population was the Pharmachild cohort at the Wilhelmina Children’s Hospital in Utrecht, The Netherlands. JIA patients aged 0 to 18 years treated with weekly MTX (oral or subcutaneous) and with available plasma samples in the biobank were included. A baseline sample had to be available within 1 to 20 weeks after first start of MTX therapy. A follow up sample had to be available within 9 to 15 months after start, reflecting one year of MTX use. The interval between the two samples had to be at least 20 weeks to ensure two independent moments of adherence monitoring. In case more samples were available within the defined periods, the sample accompanied with a completed JAMAR, and closest to MTX start or to 12 months of therapy was selected. A patient was excluded when therapy with MTX was temporarily not in use during blood sampling according to information documented in the electronic health records (e.g., MTX paused due to infection).

### Primary and secondary endpoints

The primary endpoint was the percentage of selected JIA patients adherent to MTX at start of MTX therapy and after one year of use, according to our MTX assay. See also paragraph *Definition of adherence*. Secondary endpoints were the association between adherence according to the MTX assay and self-reported adherence in the JAMAR, and the association between adherence and the use of concomitant antirheumatic treatment with biologics.

### Methotrexate assay specifications

The bioanalysis of the samples was performed by the Division Laboratory and Pharmacy of the University Medical Center Utrecht, The Netherlands using an ultrasensitive and validated bio-analytical LC-MS/MS method for MTX and 7-OH-MTX in plasma. The samples were injected (25 µL) on a Avantor Alltima HP C18-EPS 3 μm, 150 × 2.1 mm column (column temperature 30 °C). Detection was performed on a Thermo Scientific TSQ Altis triple quadrupole mass spectrometer with positive ionisation, spray voltage 4000 V, sheath gas 40 Arbitrary units (arb) and auxillary gas 15 arb. The ion transfer tube temperature was 325 °C and the vaporiser temperature was 350 °C. Ions monitored in the selected reaction monitoring mode were mass-to-charge ratio (m/z) 455 > 308 for MTX, m/z 459 > 312 for [^13^C,^2^H_3_]-methotrexate with a collision energy (CE) of 24 V and radio frequency (RF) of 70 V, m/z 471 > 324 for 7-OH-MTX and m/z 475 > 328 for [^13^C,^2^H_3_]-7-OH-MTX with a CE of 12 V and RF 54 V.

The precision values (coefficient of variation, CV) for MTX were 3.2%, 1.6% and 1.6% at concentrations of 0.16 nM, 0.44 nM, and 1.60 nM, respectively. For the metabolite 7-OH-MTX, the precision values were 7.4%, 6.0%, and 8.7%, at the same concentrations respectively. The assay had a lower limit of quantification (LOQ) of 0.02 nM (CV 8.3%) for MTX and 0.16 nM for the metabolite 7-OH-MTX, which are the lowest LOQs for MTX published so far [11, 12]. Recovery over a concentration range of 0.16 to 1.60 nM was 91.5 to 93.9% and 105.2 to 114.8% for MTX and 7-OH-MTX, respectively. This ultrasensitive method enabled us to detect the metabolite 7-OH-MTX during the entire dosing interval and to adjust the adherence limits for detection of true-positive adherence in > 95% of patients.

### Definition of adherence

Adherence was defined as a plasma concentration of MTX above the adherence limit, corresponding with dose and time after administration (Table 1). These adherence limits originate from Bluett et al., aiming to detect adherence at 7 days (t = 168 h) for a weekly MTX dose of ≥ 5 mg [11]. These limits were further refined to assess adherence < 168 h and were found suitable in children as well, according to the information from the study of Skoglund et al. [12] In case the moment of last MTX administration was unknown, the adherence limit of t = 168 h was taken. At each sampling point, JIA patients with MTX plasma concentrations above the adherence limit were categorised as adherent, whereas patients with concentrations below the adherence limit were categorised as potentially non-adherent. The metabolite 7-OH-MTX was additionally measured as a surrogate parameter for adherence to MTX and data from the JAMAR were used as self-reported adherence.

**Table 1:** MTX dose and corresponding MTX adherence limits (nM)^11^

	Adherence limits MTX concentration (nM)		
MTX dose (mg/week)	T = 168 h	T < 168 h	
5	0.1	< 48 h: ≥ 48 h - ≤96 h: > 96 h:	0.2 0.15 0.1
7.5	0.15	≤ 36 h: > 36 h - <96 h: ≥ 96 h - <144 h: ≥ 144 h:	0.5 0.25 0.2 0.15
10	0.2	≤ 36 h: > 36 h - ≤144 h: > 144 h:	0.5 0.25 0.2
12.5	0.25	< 48 h: ≥ 48 h:	0.5 0.25
15	0.25	≤ 96 h: > 96 h:	0.5 0.25
17.5	0.25	≤ 144 h: > 144 h:	0.5 0.25
20	0.25	≤ 48 h: > 48 h - ≤144 h: > 144 h:	0.75 0.5 0.25
22.5–25	0.5	< 72 h: ≥ 72 h:	0.75 0.5

MTX, methotrexate

### Juvenile arthritis multidimensional assessment report

Patients and parents/caregivers were asked to complete a JAMAR questionnaire in advance of every outpatient clinic visit to the pediatric rheumatologist as part of routine clinical care.

This questionnaire focusses on the overall wellbeing of the JIA patient and contains questions about medication intake. The questions “Do you take your medicines at the times stated by the physician?“, accompanied by the explanation why the medicines were not taken at the stated times and “What medicine is the hardest to administer at fixed times” were used to assess self-reported adherence if the JAMAR was completed within a range of 14 days around the blood sampling.

### Statistical analysis

Descriptive statistics were used to characterise the study population. Adherence to weekly MTX shorty after initiation of therapy and around one year of MTX use were expressed as percentages. Also a sub-analysis was performed in the part of patients with a known day of administration and a sensitivity analysis with an adherence limit of t = 168 for all patients. Mann-Whitney U test was used to compare patient ages between adherent and possibly non-adherent patients. Fisher’s Exact tests were used to test the association between adherence according to the MTX assay and self-reported adherence in the JAMAR, and the association between adherence and the use of concomitant antirheumatic treatment with biologics. Two-sided, statistical significance was established with an alpha of 0.05. Statistical analysis was conducted with IBM SPSS statistics version 26.0.0.1.

## Results

### Characteristics of the study population and details of treatment

A total of 43 JIA patients met the criteria of the sample selection. The median age at start of MTX was 11 years (range 1 to 17 years) and 25 (58%) patients were female. The route of MTX administration was mostly oral intake of a tablet (86%). Five patients (12%) used MTX subcutaneous injection fluid orally and 1 (2%) patient used MTX subcutaneously. For all patients, the route of administration did not change during MTX treatment. The weekly MTX dose ranged from 5 mg to 25 mg with a median of 12.5 mg. At baseline, 5 (12%) patients had concomitant treatment with a biological. This number increased to 17 (40%) patients after one year. See Table 2 for patient characteristics at start of MTX therapy and further details of JIA treatment. In 18 and 17 patients the day of MTX administration was known during baseline and follow up sampling, respectively.

**Table 2:** Patient characteristics and details of treatment

	JIA patients (*n* = 43)	
Patient characteristics at start of MTX treatment ^a^		
Female (n)	25 (58,1%)	
Age (y)	11 (6–14)	
Height (cm)	145 (112–161)	
Weight (kg)	35.3 (21.0-47.7)	
BMI	16.6 (15.1–18.7)	
BSA (m^2^)	1.19 (0.83–1.45)	
Dose BSA-normalised (mg/m^2^)	11.3 (10.5–13.6)	
Details of treatment ^a^		
Total MTX duration (days)	668 (461–896)	
	**Baseline**	**Follow up**
MTX dose (mg) ^a^	12.5 (10–15)	12.5 (10–20)
Route of administration (n)		
- Subcutanous - Oral, tablet - Oral, inj. fluid	1 (2.3%) 37 (86.0%) 5 (11.6%)	1 (2.3%) 37 (86.0%) 5 (11.6%)
Comedication (n)		
- Adalimumab - Anakinra - Etanercept - Canakinumab	3 (7.0%) 1 (2.3%) 1 (2.3%) 0 (0.0%)	12 (27.9%) 1 (2.3%) 3 (70%) 1 (2.3%)

^a^ median (interquartile range); BMI, body mass index; BSA, body surface area; inj, injection; JIA, juvenile idiopathic arthritis; MTX, methotrexate

### Methotrexate and 7-hydroxy-methotrexate concentrations

At baseline, there was a median time period of 10.7 (range 1.6 to 19.9) weeks between the start of MTX and the date of sampling. The median baseline concentration was 0.34 (range 0.14 to 197) nM for MTX and 1.58 (range 0.31 to 285) nM for 7-OH-MTX. At follow up, the median interval between the start of MTX and the date of follow up sampling was 11.7 (range 9.0 to 14.5) months. The median concentration was 0.30 (range 0 to 13.5) nM for MTX and 1.08 (range 0 to 96.5) nM for 7-OH-MTX after one year. See Table 3 for detailed information on the MTX and 7-OH-MTX concentrations.

**Table 3:** Sampling details and MTX and 7-OH-MTX concentrations

Details of sampling ^a^	Baseline	Follow up
Time to sampling since MTX start (days)	75 (59–96)	358 (315–395)
MTX concentration (nM)	0.34 (0.28–0.88)	0.30 (0.24–0.50)
Dose-normalised MTX (nM/mg)	0.03 (0.02–0.07)	0.03 (0.02–0.06)
7-OH-MTX concentration (nM)	1.58 (0.76–7.51)	1.08 (0.59–2.91)
Dose-normalised 7-OH-MTX (nM/mg)	0.12 (0.06–0.75)	0.09 (0.04–0.29)
MTX:7-OH-MTX ratio	3.52 (2.93–7.80)	4.12 (2.48–6.35)

^a^ median (interquartile range); 7-OH-MTX, 7-hydroxy-methotrexate; MTX, methotrexate

### Adherence according to the methotrexate assay

At baseline, 38 (88%) patients had a MTX concentration above the corresponding adherence limit. After one year, this decreased to 33 (77%) patients. In the sub-analysis performed in the 40% of patients with a known day of administration, MTX adherence percentages were 83% at baseline and decreased to 59% after one year of use. The sensitivity analysis showed adherence percentages of 91% and 84% at baseline and after one year, respectively.

**Fig. 1:**
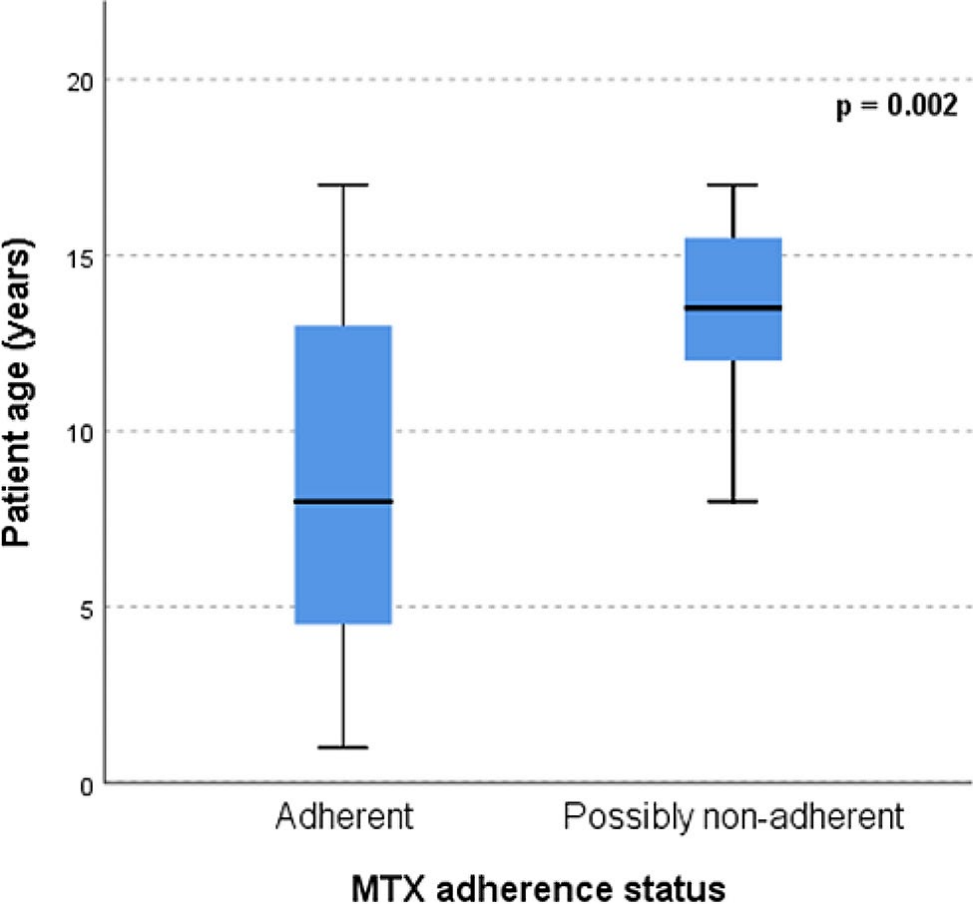
Comparison of patient ages between adherent and possibly non-adherent children

The characteristics of the patients with MTX concentrations below the corresponding adherence limits are shown in Table 4. There were 3 patients with MTX concentrations below the adherence limit at both sampling points, including one patient with even undetectable MTX and 7-OH-MTX concentrations after one year. Possibly non-adherent patients had a higher age (*p* = 0.002), see Fig. 1.

**Table 4:** Characteristics of the patients with MTX concentrations below the adherence limit

Patient	Sex	Age (y)	Time after start MTX (days)	Dose (mg)	Form	Biological	Information JAMAR (child)
Baseline							
1*	M	13	42	7.5	Tablet	None	No data
2**	F	17	63	20	Tablet	None	Adherent
3***	M	15	91	20	Tablet	None	Adherent
4	M	14	62	15	Tablet	None	No data
5	F	16	70	20	Tablet	None	No data
Follow up							
1*	M	13	343	7.5	Tablet	Adalimumab	Non-adherent
2**	F	17	378	20	Tablet	None	Adherent
3***	M	15	383	20	Tablet	None	Non-adherent
4	F	16	357	20	Tablet	None	Adherent
5	F	13	273	12.5	Tablet	None	No data
6	F	11	315	10	Tablet	Etanercept	No data
7	F	8	389	10	Tablet	None	No data
8	M	13	349	15	Tablet	None	Adherent
9	M	11	427	12.5	Tablet	None	No data
10	F	14	294	20	Tablet	None	Adherent

*/**/*** Patients with MTX concentrations below the adherence limit at both sampling points; JAMAR, juvenile arthritis multidimensional assessment report; MTX, methotrexate

### Juvenile arthritis multidimensional assessment report

The JAMAR was completed by 11 children and 8 parents/caregivers at baseline sampling. At follow up sampling after one year, the JAMAR was completed by 13 children and 16 parents/caregivers. All children at baseline and all parents/caregivers at both sampling points reported no signs of non-adherence. At follow up sampling after one year, three children reported non-adherence. Two of them reported difficulties with the use of MTX at fixed times; the other child reported to take no medication (including MTX) at all.

### Association between adherence and the juvenile arthritis multidimensional assessment report

Two patients reported MTX as the drug to be the hardest to administer at fixed times (Table 4). They both had MTX concentrations below the adherence limit (including one with undetectable MTX and 7-OH-MTX concentrations). Another child reported to use no medication at time of sampling, however, this child had detectable MTX and 7-OH-MTX concentrations above the adherence limit. We could not find an association between MTX adherence after one year and available self-reported information in the JAMAR (Fisher’s Exact, *p* = 1.00), see Supplementary Table S1.

### Association between adherence and concomitant use of biologicals

Two potentially non-adherent patients had concomitant therapy with biologics. We could not find an association between MTX adherence after one year and concomitant use of biologic treatment (Fisher’s Exact, *p* = 0.27), see Supplementary Table S2.

## Discussion

Adherence to weekly MTX therapy in our JIA population was 88% shortly after initiation of MTX therapy and 77% after one year, according to our ultrasensitive LC-MS/MS MTX assay. These findings are in line with adherence percentages described in medical literature [4, 5]. Teenagers were more at risk for non-adherence (*p* = 0.002, Fig. 1). A negative association between age and adherence was also earlier described [5]. 

There was no significant association detected between adherence according to the MTX assay and self-reported adherence in the JAMAR questionnaire, nor with the use of concomitant biologics. A potential explanation for this could be the small sample size. Only a relatively small part of children with materials in the Pharmachild biobank had plasma samples available after one year of MTX use. On the other hand, this could reflect a cohort that was relatively tolerable of MTX with less risk of non-adherence issues. MTX intolerance develops mostly within the first year of use and none of the patients in our cohort were tapering MTX or switched the route of administration (as coping mechanism of intolerance) [8, 13]. According to our protocols, JIA patient take MTX as long as it is effective as monotherapy in an as high as tolerable dose. When additional (biological) medication is started, MTX is ideally not tapered, because of its dose-dependent inhibiting effect on the forming of anti-drug antibody against biologicals.

A potential limitation of the use of MTX in plasma for adherence assessment is that it only reflects short term adherence. MTX has a relatively short half-life and there is a risk of ‘white coat compliance’ with a patient taking MTX shortly before a doctor’s appointment. In our study, however, patients were not aware of MTX adherence monitoring at the time of blood sampling. Moreover, quantification of 7-OH-MTX can provide additional information about the moment of medication intake and metabolism, regarding for instance the MTX: metabolite ratio. With our ultrasensitive MTX assay, we were able to detect the metabolite 7-OH-MTX during the entire dosing interval. This in contrast with other published assays [11, 12]. No discrepancies were seen in 7-OH-MTX formation in our study population.

A method in favour of long-term adherence monitoring is measurement of intracellular methotrexate polyglutamate (MTXPG). MTXPG concentrations have been demonstrated to be a potential surrogate biomarker of adherence to long-term therapy in children with JIA and juvenile dermatomyositis [14]. However, analysis of MTXPG in red blood cells is complicated and expensive and therefore, less suitable for routine clinical monitoring. Moreover, information on adherence can only be assessed regarding changes or fluctuations in MTXPG concentrations over time. This makes quantification of MTX concentrations in plasma the most feasible method for physicians to assess adherence to low-dose MTX, even with one-time sampling. Besides, lack of adherence to MTX is not unique for JIA patients. It is also a major issue in childhood acute lymphatic leukemia, now enabling us to monitor MTX adherence in this population as well [15]. 

A second caveat is that the MTX pharmacokinetics may differ between children and adults. Children seem to tolerate higher doses than adults, caused by a reverse age-dependant elimination of MTX [16, 17]. Dose-normalised concentrations for MTX are also lower in children than in adults [17]. Therefore, the applied adherence limits established in adult RA patients might be not fully applicable for the use in children. Regarding these limits, Bluett et al. suggested an adherence limit of 0.1 nM to be sufficient to detect adherence at 7 days for a weekly dose of ≥ 5 mg [11]. Nonetheless, Skoglund et al. also showed a 7-day MTX level of > 0,1 nM in 94% (16 out of 17) children with acute ALL on low-dose MTX maintenance therapy [12]. No patients had concentrations < 0.5 nM at 48 h after the last dose. Therefore, we considered the defined adherence limits of Bluett in adult RA patients suitable in children as well.

Noteworthy, a MTX concentration below the adherence limit may still be caused by alterations in a patient’s MTX pharmacokinetics, such as malabsorption or a faster metabolism. The bioavailability of MTX is highly variable (range 28–94%) and this may be influenced by food to some extent [18–21]. Therefore, non-adherence cannot be stated with certainty in patients with detectable MTX concentrations below the corresponding adherence limits. However, we believe that the influence of variability in absorption, due to food for instance, on the interpretation of the results in our study is minimal. The adherence limits (Table 1) are not dependent on (time to) peak concentrations (first limits are < 36 h and 48 h) and were defined to detect adherence in > 80% of compliant patients. Therefore, measurement of MTX concentrations in plasma is a suitable and objective method to further explore a case of existing suspicion of non-adherence. For instance, due to an unexplained, inadequate response to MTX despite stated adherence. Undetectable MTX plasma concentrations could give rise to the suspicion of non-adherence and could be a reason for the physician to address a patients’ adherence to therapy in order to improve it [9, 11, 15]. 

A last point of discussion is that the day of MTX administration was unknown in 60% of our patients, due to the retrospective nature of the data. In this case, we used the adherence limit of t = 168 h, which may have overestimated adherence. This was confirmed by the performed sub-analysis in the 40% of patients with a known day of administration. These results suggest that true adherence percentages ranged between 83% and 88% at baseline and were between 59% and 77% after one year of use. For optimal interpretation of MTX concentrations in clinical practice, it is important to know the dose and exact day of MTX administration.

To address most of the abovementioned limitations and caveats, a prospective study would provide a solution. However, this would give also rise to the risk of ‘white coat compliance’ with a patient taking MTX shortly before a doctor’s appointment or not even wanting to participate when it is known that plasma MTX concentrations will be checked in the study. A strength of our retrospective point of view was that patients were not aware of MTX adherence monitoring at the time of blood sampling.

## Conclusions

Quantification of MTX in plasma with a sensitive LC-MS/MS assay is a suitable and objective method to assess adherence in patients using low-dose weekly MTX. The use of this method in clinical practice could be a helpful tool for physicians to refute or support suspicion of non-adherence to MTX therapy.

### Electronic supplementary material

Below is the link to the electronic supplementary material.

Supplementary Material 1

## Data Availability

All data generated or analysed during this study are included in this published article and its supplementary information files. (Remaining) patient materials are available within the scope of consent.

## Abbreviations

7-OH-MTX: 7-Hydoxy-methotrexate
Arb: Arbitrary units
CE: Collision energy
CV: Coefficient of variation
DMARD: Disease-modifying antirheumatic drug
JAMAR: Juvenile Arthritis Multidimensional Assessment Report
JIA: Juvenile idiopathic arthritis
LC-MS/MS: Liquid chromatography-tandem mass spectrometry
LOQ: Limit of quantification
MISS: Methotrexate intolerance severity score
MTX: Methotrexate
MTXPG: Methotrexate polyglutamate
RA: Rheumatoid arthritis
RF: Radio frequency
