# Supplementary material for: Adherence to low-dose methotrexate in children with juvenile idiopathic arthritis using a sensitive methotrexate assay
Source: Pediatr Rheumatol Online J. 2024 May 7;22:52. doi: 10.1186/s12969-024-00988-y (PMC11075236; doi:10.1186/s12969-024-00988-y)
Supplement: Supplementary file 1 — Supplementary Material 1 [file 12969_2024_988_MOESM1_ESM.pdf]

SUPPLEMENTARY MATERIALS

Table S1. Association between adherence and JAMAR child, after one year

	Adherent (n=7)	Possibly non-adherent (n=6)	P-value (Fishers')
Self-reported adherence issues (n)			
- Yes	2 (28.6)	2 (33.3)	1.00
- No	5 (71.4)	4 (66.7)	

Jamar, Juvenile Arthritis Multidimensional Assessment Report

Table S2. Association between adherence and concomitant use of biologicals

	Adherent (n=33)	Possibly non-adherent (n=10)	P-value (Fishers')
Concomitant biological treatment (n)			
- Yes	15 (45.5%)	2 (20.0%)	0.27
- No	18 (54.5%)	8 (80.0%)	
